# Spatial heterogeneity in root litter and soil legacies differentially affect legume root traits

**DOI:** 10.1007/s11104-018-3667-9

**Published:** 2018-05-11

**Authors:** Sirgi Saar, Marina Semchenko, Janna M. Barel, Gerlinde B. De Deyn

**Affiliations:** 1 Department of Soil Quality, Wageningen University, P.O. Box 47, 6700 AA Wageningen, The Netherlands; 2 Department of Botany, Institute of Ecology and Earth Sciences, University of Tartu, 40 Lai St, 51005 Tartu, Estonia; 3 School of Earth and Environmental Sciences, University of Manchester, M13 9PT, Manchester, UK

**Keywords:** Functional traits, Local and systemic response, Plant-soil feedback, Root litter, Soil heterogeneity, Spatial root distribution

## Abstract

**Background and Aims:**

Plants affect the soil environment via litter inputs and changes in biotic communities, which feed back to subsequent plant growth. Here we investigated the individual contributions of litter and biotic communities to soil feedback effects, and plant ability to respond to spatial heterogeneity in soil legacy.

**Methods:**

We tested for localised and systemic responses of *Trifolium repens* to soil biotic and root litter legacy of seven grassland species by exposing half of a root system to control soil and the other half to specific inoculum or root litter.

**Results:**

Soil inoculation triggered a localised reduction in root length while litter locally increased root biomass independent of inoculum or litter species identity. Nodule formation was locally suppressed in response to soil conditioned by another legume (*Vicia cracca)* and showed a trend towards systemic reduction in response to conspecific soil. *V. cracca* litter also caused a systemic response with thinner roots produced in the part of the root system not directly exposed to the litter.

**Conclusions:**

Spatial heterogeneity in root litter distribution and soil communities generate distinct local and systemic responses in root morphology and nodulation. These responses can influence plant-mutualist interactions and nutrient cycling, and should be included in plant co-existence models.

**Electronic supplementary material:**

The online version of this article (10.1007/s11104-018-3667-9) contains supplementary material, which is available to authorized users.

## Introduction

Due to their sessile lifestyle but variable environment, plants have evolved remarkable plasticity in their growth and morphology above and below ground (Fitter 1994; de Kroon and Hutchings 1995; Palmer et al. 2012). Below ground, plants experience spatial heterogeneity in nutrient supply, in density and identity of roots (alive and dead) of different species and their associated microbial communities. Many plant species are known to place their roots and prolong their residency preferentially in patches with high nutrient content (Hodge 2004; Bradford et al. 2007). Evidence is also accumulating for plasticity in root distribution and morphology to the presence and identity of living neighbouring roots (Cahill et al. 2010; Semchenko et al. 2014). However, how plants respond to spatial heterogeneity in soil legacies left behind by deceased predecessors is still poorly understood.

Plants interact with soil biota and drive specific changes in soil microbial community composition and soil functioning (Grayston et al. 1998; Garbeva et al. 2004; Schlatter et al. 2015). These changes in the microbial communities can persist in the soil after plant death and together with the decomposing roots affect subsequent plant growth via so-called plant-soil feedback (PSF) effects (Ehrenfeld et al. 2005; Hendriks et al. 2013; Mack and Bever 2014; Zhang et al. 2016). Comparative studies examining the individual effects of root litter and soil microbial legacies on succeeding plants are currently lacking, which hinders mechanistic understanding of the factors determining the outcome of plant-soil interactions (van der Putten et al. 2013). Moreover, while feedback effects on overall plant growth and survival have been extensively studied (Bever 2003), the ability of plants to modify their morphology in response to soil legacies is still poorly understood (Baxendale et al. 2014), particularly under heterogeneous soil conditions (Hendriks et al. 2015a; Wubs and Bezemer 2016).

Soil feedback effects on succeeding plants could act via litter decomposition and mineralisation and the associated changes in plant-available nutrients (Hobbie 2015). The ability of plants to forage for heterogeneously distributed nutrients and the implications of foraging precision have been extensively studied. Preferential root placement in patches with high nutrient concentrations is often achieved by changes in root biomass distribution, but also by producing more branched and thinner roots (Hutchings and John 2003; Hodge 2004; Cahill and McNickle 2011). In addition to local conditions, root proliferation in soil patches is controlled systemically by nutrient status of the whole plant (Zhang et al. 1999; Lamb et al. 2004). If root litter primarily affects plants through increased nutrient availability due to mineralisation, then responses similar to those observed for nutrient heterogeneity are expected to occur in response to litter heterogeneity. Therefore, it can be predicted that N-rich litter of legumes induces a stronger local foraging response than the more recalcitrant litter produced by slow-growing grass species.

In addition to the effects mediated by litter decomposition, soil microbial legacies involve plant pathogens and symbionts and may induce both localised and systemic responses in root systems. Due to the host specificity of both pathogenic and symbiotic plant-microbe interactions, the strongest responses are expected to occur in reaction to soil patches previously occupied by conspecifics or closely related plant species (Bever 1994; Kulmatiski et al. 2008; van de Voorde et al. 2011). Local responses of plant roots may be efficient in case of organ-specific pathogens with limited mobility, while induced systemic response is expected to be favoured in case of attack by a pathogen that is likely to spread and infect other parts of the plant (He et al. 2002; Balmer et al. 2013). Besides physiological responses, plants may increase both local and systemic investment in structural defences (Yedidia et al. 1999), leading to changes in root diameter and specific root length (Eissenstat 1992; Eissenstat and Yanai 1997). A systemic response could also be manifested in compensatory root proliferation in soil patches uninfected by pathogens in response to a localised pathogen attack elsewhere in the root system (Steinger and Müller-Schärer 1992; Hendriks et al. 2015b).

Soil microbial legacies can also affect association between plants and microbial symbionts such as nitrogen-fixing bacteria (Liu et al. 2016). The association with nitrogen-fixing bacteria within root nodules is systemically regulated by aboveground plant organs, as they maintain the balance between energetic costs and nitrogen demand (Sasaki et al. 2014). Inefficient local nodulation can also induce other parts of the root system to compensate for the perceived N limitation by increased nodulation (Laguerre et al. 2012). To date, it remains to be tested to which extent root nodule formation in legumes is responsive to litter and soil legacies of plants, and whether such responses are local or also systemic. Root litter and soils conditioned by non-legumes may reduce the abundance of nitrogen-fixing bacteria and induce local avoidance responses and/or systemic compensatory responses in the root systems of legumes, relative to soils conditioned by legumes. It has also been shown that interactions between legumes and *Rhizobium* are highly species-specific (Wang et al. 2012; Keller 2014). Therefore, variation in nodulation efficiency could also be expected in response to soil conditioned by different legume species.

While many studies have investigated microbially mediated plant-soil feedback (PSF), the relative contribution of soil microbial community *versus* root litter legacy to plant growth and belowground foraging is poorly understood, yet important in order to make PSF effects more predictable (van der Putten et al. 2013; Cortois et al. 2016). The aim of this study was therefore to investigate whether soil biota and root litter induce distinct responses in a focal legume plant at local and whole root system level, and to test if variation in these responses depends on soil inoculum and root litter identity. Specifically, the following hypotheses were tested: 1) focal legume plants exhibit localized and systemic responses in their root traits when exposed to root litter or soil inoculum from different plant species; 2) these responses vary depending on the species identity and functional group (legumes vs non-legumes) of plants that produced the litter or conditioned the soil; 3) litter nutrient content explains variation in the legume responses to root litter produced by different species.

To test these hypotheses, we conducted a split-pot experiment with *Trifolium repens* (L.) as the focal species. *T. repens* was chosen due to the importance of N-fixation in grassland ecosystems as well as the wide use of legumes as green manure and cover crops. Moreover, legumes form specialist mutualisms with N-fixing bacteria (Dassen et al. 2017) making them highly suitable for studying plant-soil feedbacks. In the split-pot experiment, *T. repens* roots were subjected to either root litter or inoculum from soils previously occupied by conspecifics or a range of other species in one compartment, and non-specific background soil without root litter in the other compartment. As a control, we grew *T. repens* in split-pots where both compartments contained non-specific background soil. This approach allowed the identification of local responses, i.e. changes in root traits appearing only in the compartment that received litter or soil inoculum, and systemic effects that also encompassed root responses in the compartment where the roots had no direct contact with litter or specific soil microbes. We predicted that *T. repens* would proliferate its roots in patches with presence of root litter of high N content and that its roots would avoid patches with microbial legacies of its own species as most conspecific soil feedbacks are negative (Kulmatiski et al. 2008; Hendriks et al. 2015a, b; Cortois et al. 2016).

## MATERIALS AND METHODS

### Conditioning phase

Soil for all treatments was collected from a semi-natural grassland site in Netherlands in spring 2013 (‘Clue’ site, Mosselse Veld, 52°04’N, 5°45’E). The soil is sandy-loam, with particle size distribution: < 2 mm, 3.4%; 2–63 mm, 17.3%; > 63 mm, 79.7%, pH H_2_O 6.4 and %OM 4.5 (Van der Putten et al. 2000; Bezemer et al. 2010). The soil was sieved (8 mm) and stored at 4 °C. Pots with a volume of 2 l were filled with a soil mixture consisting of 85% sterilised soil (25 kGray) and 15% fresh soil to reintroduce the natural soil microbial community. The bulk of the soil was sterilised to eliminate weeds and insect herbivores. Reinoculation of sterilised soil with living soil inoculum ensured the soil microbial community adaptation to the conditioning plant species (Francioli et al. 2016). The soil was subsequently conditioned by each of seven species naturally occurring in temperate semi-natural grasslands on sandy soil: 3 grasses (*Lolium perenne, Festuca rubra* and *Arrhenatherum elatius)*, 3 legumes (*Trifolium repens, Trifolium pratense* and *Vicia cracca*) and a forb (*Cichorium intybus*). The seeds were purchased from specialised companies: *T. pratense* and *C. intybus* from Cruydt-Hoeck (Groningen, Netherlands), *L. perenne* and *T. repens* from Agrifirm (Apeldoorn, Netherlands), *V. cracca*, *F. rubra* and *A. elatius* from Emorsgate (Norfolk, UK). Before planting, the seeds were surface sterilised with 10% household bleach solution for 30s, rinsed repeatedly with tap water and germinated on autoclaved sand. Two weeks after germination, eight seedlings of each species were planted as a single individual per pot and were grown in a greenhouse for 12 weeks with additional illumination to achieve 16 h of daylight and with a day:night temperature cycle of 21:16 °C to create eight independent replicates of conditioned soil per plant species. At harvest, shoots were separated from roots, and the roots were removed from the soil and washed before drying at 40 °C for a week to be used as root litter in the feedback experiment. The conditioned soil was kept separate per pot, sieved (5 mm) and preserved in dark at 4 °C temperature until its use in the feedback experiment.

### Feedback phase

*Trifolium repens* L. was used as focal response plant. The feedback phase of the experiment was conducted in the greenhouse during January to May 2014. Seeds of *T. repens* were sterilised for 1 min in 10% household bleach solution, rinsed with tap water and sown in 200 ml plastic cups in a soil mixture consisting of 90% sterilised and 10% non-sterilised field soil (85.8 ± 5.0 P-PO_4_, 270.6 ± 15.0 N-NH_4_, 91.4 ± 15.0 N-(NO_3_ + NO_2_) mg per kg soil dry weight). After eight weeks the plants were prepared for transplanting, their roots rinsed with tap water and the main root of each plant clipped to 2 cm length. The *T. repens* plants were then transplanted into split-pots such that the roots were distributed equally between the two compartments of the split-pot. Split-pots were constructed by gluing together two 11x11x12 cm plastic pots creating one experimental unit with two compartments (Supplementary material, Fig. S1). A shallow wedge was cut in the partitioning wall so that each plant could be placed on the top of the partitioning wall of the two compartments. The soil surface was covered with a thin layer of autoclaved sand to prevent moss and algal growth.

Plants were subjected to two treatments: a soil microbial legacy and a litter legacy treatment. We did not combine the microbial and litter legacy treatments in a factorial design as each treatment was represented by seven levels, so that a full-factorial design would result in a vast number of combinations, measurements and comparisons. Instead, this experiment treated microbial and litter legacies separately with the aim of assessing their individual effects on plant foraging. For the soil microbial legacy treatment one of the compartments was filled with an unconditioned soil mixture consisting of 10% unconditioned field inoculum and 90% sterilised soil (same mixture as for seedling rearing) and the other compartment inoculated with soil conditioned with one of the seven plant species (10% conditioned soil mixed with 90% sterilised soil). The control treatment received the unconditioned soil mixture in both compartments. For each treatment with soil of different plant species, four independent soil pools conditioned by each plant species were prepared by pooling soils from two out of eight pots of same species from the conditioning phase. This was done to match the litter legacy treatment where litter was pooled in the same combinations to ensure sufficient litter quantity.

For the root litter legacy treatment, both compartments were filled with the unconditioned soil mixture (10% unconditioned field soil and 90% sterilised soil) and with one of the compartments containing a litterbag with root litter of one of the seven plant species. In the control treatment, an empty litterbag was inserted in one of the compartments. The root litter was composed of fine roots (diameter < 2 mm), dried at 40 °C and cut to 5 mm long pieces. Each litterbag contained 0.5 g of dried root litter, which was obtained by pooling roots of two conspecific individuals to obtain enough root litter for filling two litterbags. This resulted in four independent root litter pools per root litter species, each containing roots of two individuals of the same plant species. The same combinations of pots were used for pooling root litter as those used for pooling the soil inocula. The litterbags were made of polyester fabric (mesh size <0.05 mm, litter bag size 70 × 70 mm). Litterbags were placed vertically with outer edge at a distance of 2 cm from the pot border and upper edge slightly covered with sand (Supplementary material, Fig. S1).

There were eight replicates per treatment per species resulting in a total of 64 pots for the soil inoculum treatment and 64 pots for the root litter treatment. Each root litter and soil inoculum pool of each conditioning species was represented by two pots. The pots were placed in a greenhouse in a randomised block design with eight blocks. Three weeks after planting, leaves and protruding stems were clipped to 2 cm height to prevent clonal spread into neighbouring pots and stimulate root growth. The plants were kept in a greenhouse at 16:21 °C night:day cycle with additional lighting to achieve 16 h day length from beginning to the end of experiment during 16 weeks. The pots were watered daily to 60% water holding capacity by adjusting the weight loss of the pot due to evapotranspiration.

### Measurements

We determined the concentrations of N, P and C on a subsample of each root litter pool before the start of the feedback experiment as described in Saar et al. (2016). The subsamples of dried roots were ground, then mineralised using H_2_SO_4_/H_2_O_2_/Se wet digestion (Novozamsky et al. 1983), and subsequently analysed for total N and P content using Segmented Flow Analyses (SFA). Another subsample of ground roots was used to quantify the C and N concentration in the litter using a CN elemental analyser (LECO, Germany). Due to limited litter availability for this CN analysis, there were 3 instead of four replicates for *L. perenne*, *T. pratense* and *V. cracca* and two for *T. repens*.

Plants were harvested eight weeks after planting. Aboveground biomass was removed and roots washed out separately from the two compartments. A representative root sample was preserved in 50% alcohol from each compartment which was used for scanning and quantification of specific root length (SRL; scanned root length/scanned root dry mass), average root diameter, root branching frequency (the number of root tips per unit of scanned root length) and the number of nodules per gram of dry root mass using WinRhizo Pro 2013e to analyse the scanned images. Shoots, scanned and remaining roots were dried at 40 °C for 48 h and weighed separately.

### Statistical analysis

Data analyses were performed using R (version 3.1.2). All response variables were ln-transformed prior to analysis to satisfy model assumptions. The effects of litter addition and soil inoculum from different species on the traits of the response plant *T. repens* were tested separately using linear mixed models (package *lme4* (Bates et al. 2014)) with litter or soil origin (seven conditioning species), compartment (treatment or untreated) and their interaction as fixed factors, pot nested within block as random factors, and root biomass, root diameter, SRL, branching frequency and the number of nodules per gram of root dry mass of *T. repens* as response variables. The same model but without the factor compartment was used to analyse data on total shoot biomass. The inclusion of litter and soil inoculum pool as additional random factors did not significantly improve model fit and produced nearly identical estimates for fixed effects; these random factors were hence excluded from the final analyses. Local response is signified by a change in root traits in the treated compartment (with a specific soil inoculum or root litter), while significant changes in both compartments or in the untreated compartment only (not directly exposed to a specific soil inoculum or root litter) signifies a systemic response.

To test the effects of plant functional group on root traits (Hypothesis 2), linear mixed models were used with functional group (legume or non-legume) and compartment as fixed factors, and pot nested within block and soil or litter species as random factors. To test the role of litter properties (Hypothesis 3), we calculated response ratios as ln(treated compartment/control compartment), averaged response ratios by litter pool (there were four litter pools per species distributed among 8 pots, therefore averaging across 2 pots) and used linear models with the mean response ratio as a response variable, litter N, P or CN as fixed factors and root litter species as a random factor.

To test if localised plant responses to litter deviated significantly from the control treatment with an empty litter bag, response ratios were calculated as ln(treated compartment/untreated compartment) and linear mixed models were used with litter origin (seven species plus the control) as a fixed factor and block as a random factor. Species-specific response ratios were compared to the empty-litterbag control treatment as a reference level using t-tests. To test for the significance of overall localised responses (main effect of compartment in the models described above) compared to the control treatment with an empty litterbag, Helmert contrasts and t-test were used to compare the response ratios of all seven conditioning species combined to the response ratio of the control treatment. To test for the significance of a localized response to soil conditioning, the mean trait value in the compartment with inoculum was compared to the mean trait value in the control treatment with unconditioned soil in both compartments using t-tests as described above. To test if systemic plant responses deviated significantly from the control treatments (empty litterbag or unconditioned soil in both compartments), linear mixed models were used with soil or litter origin (seven species plus the control) as a fixed factor and pot nested within block as random factors and ln-transformed trait as a response variable. Helmert contrasts and t-test were used to compare the mean trait value of all seven conditioning species combined to the mean of the control treatment. All Helmert contasts and t-tests were performed using *lmer* models and *summary* function in package *lme4* (Bates et al. 2014).

## RESULTS

### Plant responses to soil legacies

#### Plant biomass

**Fig. 1:**
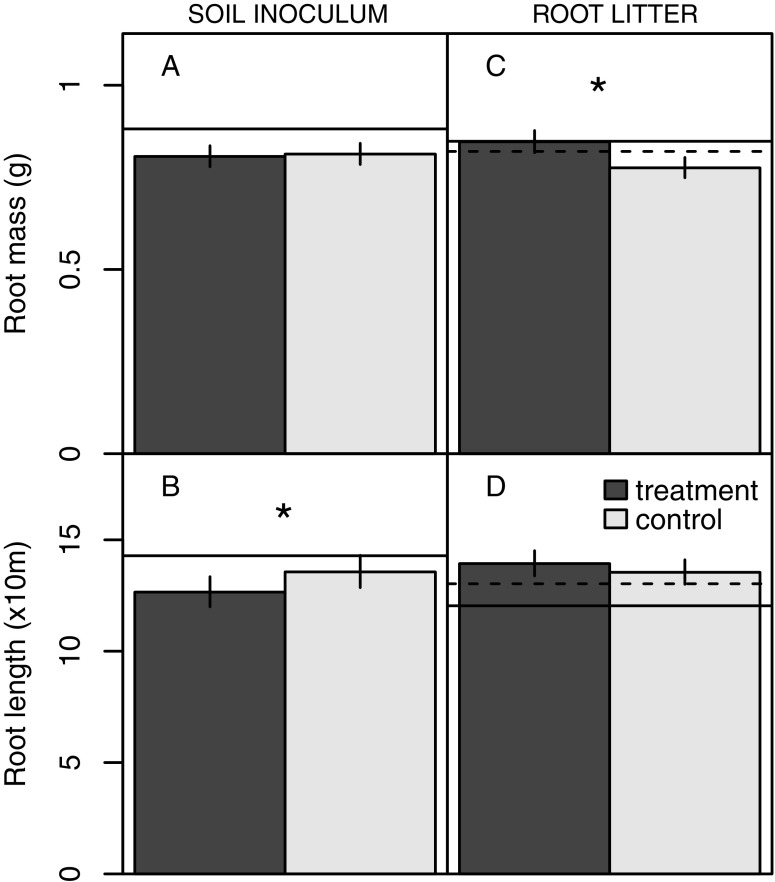
The general effect of soil inoculum (A-B) and root litter addition (C-D) on root dry mass and total root length of *Trifolium repens*. The plants were grown in split-pots with one compartment treated (dark grey) with plant species specific soil inoculum (A-B) or root litter (C-D), and the other compartment incoculated with unconditioned soil (untreated compartment; light grey). In the soil inoculum graphs (A-B) the solid line indicates the mean trait value for the control treatment with both compartments filled with unconditioned soil mixture. In the root litter graphs (C-D) the dashed and solid lines indicate mean trait values for the control treatment with one compartment filled with unconditioned soil only and the other compartment containing unconditioned soil and an empty litterbag, respectively. Asterisks indicate significant differences between untreated and treated compartments (*P* < 0.05, Tukey test)

Total aboveground biomass of *T. repens* was not affected by soil inoculum from the different plant species (F_6,42_ = 0.82, *P* = 0.5599) and similarly total root biomass of *T. repens* was not affected by soil inoculum (Fig. 1A; Table 1). Also when compared to the control the mean above- and belowground biomass across all soil inoculum treatments did not significantly differ from that of plants grown in unconditioned soil (*P* > 0.05, t-test comparing the mean of conditioning treatments to the mean of the control).

**Table 1:** Root trait responses of *T. repens* to soil inocula conditioned by seven plant species. Linear mixed models contained soil inoculum origin (seven species), compartment (inoculated *vs*. unconditioned) and their interaction as fixed factor and pot nested within block as random factors. F-values and their significance are shown. Values in bold indicate significant effects (*P* < 0.05) and marginally non-significant effects are shown in italic (*P* < 0.1); * indicates *P* < 0.05; ** indicates *P* < 0.01

Factor	df	Root mass (g)	Root length (cm)	Specific root length (cm/mg)	Root diameter (mm)	Branching frequency (tips/m)	No. nodules/ g root mass
Soil origin (S)	6,42	1.35	0.85	1.04	0.61	1.00	4.29**
Compartment (C)	1,49	0.07	4.39*	*3.56*	0.90	1.65	1.03
S × C	6,49	0.48	1.81	0.85	0.60	*2.08*	2.38*

#### Root architecture and nodulation

**Fig. 2:**
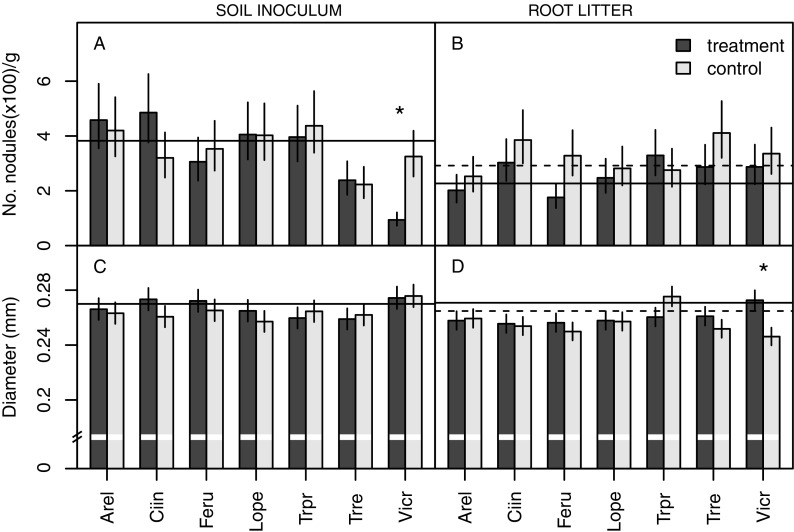
The specific effect of soil inoculum (A-B) and root litter addition (C-D) originating from different plant species on the number of nodules produced per gram of dry root mass and root diameter of *Trifolium repens*. The plants were grown in split-pots with one compartment treated (dark grey) with specific soil inoculum (A-B) or specific root litter (C-D) and the other compartment inoculated with unconditioned soil with no litter addition (untreated compartment, light grey). In the soil inoculum graphs (A-B), the solid line indicates the mean trait value for the control treatment with both compartments filled with unconditioned soil mixture. In the root litter graphs (C-D), dashed and solid lines indicate mean trait values for the control treatment with one compartment filled with unconditioned soil only and the other compartment containing unconditioned soil and an empty litterbag, respectively. Conspecific treatments (Trre) are indicated in bold. Asterisks indicate significant differences between untreated (light grey) and treated (dark grey) compartments (*P* < 0.05, Tukey test). Specific soil inoculum and root litter from: Arel – *Arrhenatherum elatius*, Ciin – *Cichorium intybus*, Feru – *Festuca rubra*, Lope – *Lolium perenne*, Trpr – *Trifolium pratense*, Trre – *Trifolium repens*, Vicr – *Vicia cracca*

In contrast to plant biomass, *T. repens* showed local belowground responses to the soil microbial legacy from other plants, as indicated by the altered root architecture traits (Table 1, Figs. 1, and 2). Total root length displayed a significant local response to soil conditioning independent of species identity, as evidenced by less root length in the treated compartment compared to the untreated compartment (Table 1; Fig. 1B). The root length in the compartments treated with conditioned soil was however not significantly different from the control treatment with unconditioned soil in both compartments (*P* = 0.2305, t-test). A specific soil conditioning effect was observed on local nodulation (Table 1). Nodulation was strongly suppressed (by 71%) in the compartment containing soil conditioned by *Vicia cracca* when compared with nodulation of roots in the untreated compartment (Fig. 2A). The production of root nodules in the *V. cracca* soil was also significantly lower compared to the unconditioned control treatment (*P* = 0.0298, t-test). No significant localised responses in nodule numbers were observed in response to other conditioned soils (significant interaction between soil legacy and compartment in Table 1). The local response in nodule number tended to differ between functional groups: nodulation in legume soil was lower than in soil conditioned by non-legumes, but this effect was only marginally significant and probably due to the strong response to *V. cracca* soil inoculum (interaction between functional group and compartment, F_1,54_ = 3.77, *P* = 0.0574). No significant systemic responses to soil inoculation were found for any of the measured plant traits (no significant effect of soil origin in Table 1), except for the trend towards lower nodulation in both treated and untreated compartments when *T. repens* plants were exposed to soil conditioned by conspecifics (*P* = 0.1150, t-test, 40% lower nodulation in plants exposed to soil conditioned by conspecifics compared to plants in the control treatment with unconditioned soil in both compartments).

### Plant responses to root litter

#### Plant biomass

The local presence of root litter of the various plant species did not affect the total above- and belowground biomass of *T. repens* (F_6,42_ = 0.28, *P* = 0.9428 and main effect of litter origin on root mass in Table 2, respectively). The mean above- and belowground biomass across all root litter treatments did not significantly deviate from that recorded for the control treatment with empty litterbags (*P* > 0.05, t-test). In contrast, root litter presence did affect the spatial distribution of the root biomass (significant main compartment effect in Table 2, Fig. 2C). *T. repens* had more root biomass in the treated compartment with litter compared to the untreated compartment, irrespective of the root litter species. However, this local root biomass response was not significantly different from the empty litterbag control treatment (*P =* 0.5318, t-test comparing response ratios).

**Table 2:** Root trait responses of *T. repens* to root litter from seven plant species. Linear mixed models contained soil inoculum origin (seven species), compartment (with *vs*. without litterbag) and their interaction as fixed factor and pot nested within block as random factors. F-values and their significance are shown. Values in bold indicate significant effects (*P* < 0.05) and marginally non-significant effects are shown in italic (*P* < 0.1); * indicates *P* < 0.05; ** indicates *P* < 0.01

Factor	df	Root mass (g)	Root length (cm)	Specific root length (cm/mg)	Root diameter (mm)	Branching frequency (tips/m)	No. nodules/ g root mass
Litter origin (L)	6,42	0.71	0.80	0.29	0.67	1.04	1.14
Compartment (C)	1,49	8.61**	0.35	*2.94*	2.36	<0.01	9.64**
L × C	6,49	0.35	0.45	1.20	3.08*	1.10	0.85

#### Root architecture and nodulation

There was a significant local response in nodule numbers to litterbag presence, irrespective of the identity of the root litter (significant main compartment effect in Table 2). Fewer nodules were produced in most treated compartments (Fig. 2B). However, this response was not significantly different from that observed in the control treatment with an empty litterbag (*P* = 0.9748, t-test comparing response ratios of the pot half with and without litterbag). Root diameter also showed a significant response to litter presence and this effect was dependent on root litter identity (significant interaction between litter species and compartment in Table 2). Average root diameter was significantly reduced in the untreated compartment compared to the treated compartment with *V. cracca* litter (Fig. 2D), and differed significantly from the empty-litterbag control (*P* = 0.0399, t-test comparing response ratios). Variation in root diameter responses to the different root litter species could not be explained by the functional group (non-significant interaction between the effects of functional group and compartment, F_1,54_ = 0.75, *P* = 0.3905). The root diameter response was also not significantly related to litter N (F_1,5_ = 0.5932, *P* = 0.474) or C content (F_1,20_ = 0.4009, *P* = 0.5335) or to litter C:N ratio (diameter F_1,5_ = 0.0067, *P* = 0.9378).

## DISCUSSION

The results of this study demonstrate that spatial heterogeneity in root litter distribution and soil microbial legacy can trigger localised and systemic response in the root systems of *T. repens*. Specifically, we found root length to decrease locally in response to conditioned soil, and root biomass to increase in the vicinity of root litter independent of soil inoculum or litter identity. In addition, plants modified root nodulation and root diameter in response to soil biotic and litter legacy of another legume species. While many plant-soil feedback studies demonstrated poorer plant growth on soil conditioned by conspecifics compared to soil conditioned by other species (Klironomos 2002; Wubs and Bezemer 2016), no such trend was detected in this study. Instead, *T. repens* experienced the most negative feedback from soil biota and root litter when these were accumulated by another closely related species *V. cracca*.

### Soil conditioning and spatial effects

Plants responded locally to soil conditioned by different grassland species by reducing root length in the compartment inoculated with conditioned soil relative to the root length in the compartment with control (unconditioned) soil (Fig. 1B). However, we did not detect stronger effects on plant traits from soil conditioned by conspecifics as compared to soil conditioned by heterospecifics, which is in contrast with findings of species-specific feedback effects on plant biomass shown in previous studies (Bever 1994; Kulmatiski et al. 2008; van de Voorde et al. 2011). Localised suppression of root length in soils conditioned by species monocultures independent of species identity may be due to monoculture soils accumulating more generalist pathogens than the control inoculum collected from a grassland occupied by a mixture of species. Overall, no significant decline in plant biomass was detected in response to inoculation with soil conditioned by different species. This finding may indicate that *T. repens* generally exhibits weak plant-soil feedback as has been shown for legumes in previous studies (Cortois et al. 2016). Also, *T. repens* could have limited the negative feedback on overall plant growth by maintaining root functioning in the control compartment with inoculum from a semi-natural grassland, despite growth reduction in the compartment with species-specific inocula (Hendriks et al. 2015b).

In contrast to some other plant trait responses, a clear localised and species-specific response to soil legacy was observed in the number of root nodules produced per unit of dry root mass, which was significantly lower in the compartment inoculated with soil conditioned by *Vicia cracca* (Hypotheses 1 and 2 partly supported). *Rhizobium* strains can form highly species-specific associations with plants (Van Berkum et al. 1995; Wang et al. 2012). It is therefore possible that *V. cracca* accumulated specific rhizobial strains that were not readily forming symbiosis with *T. repens*. Moreover, closely related plant species are more likely to be susceptible to the same fungal pathogens (Gilbert and Webb 2007). If closely related legumes show host-specificity in their associations with mutualists but share the same pathogens, this could result in negative feedback from heterospecific legume plants, but more neutral feedbacks from less related non-legume species as observed in this study. Moreover, pathogenic fungi can locally suppress symbiotic rhizobia (Liu et al. 2016), which could result in the reduction of nodulation in soil conditioned by closely related species. In addition, there was a tendency for a systemic response to inoculation with soil conditioned by conspecifics: *T. repens* produced lower numbers of nodules in both soil compartments when in contact with *T. repens* soil inoculum (marginally non-significant effect). Such systemic response would indicate that soil biota accumulated in the rhizosphere of conspecifics can have a negative impact on the formation of symbiosis locally as well as at the level of the whole plant through altered plant signalling.

### Consequences of litter effects

Root litter addition caused a localised reduction in root biomass and nodule production independent of litter species identity. However, these responses were not significantly different from the response to an empty litterbag in the control treatment. This suggests that physical rather than chemical processes were driving the observed response. It has been shown that plants can respond to physical obstacles in the soil with the redirection of root growth into soil with fewer obstructions, as well as by reduction in root branching and specific root length in areas with high density of obstructions (McConnaughay and Bazzaz 1992; Falik et al. 2005; Semchenko et al. 2008). As plants in this study produced more root biomass but similar root length in the compartment with a litterbag (Fig. 1), it is likely that *T. repens* perceived the litterbags as obstructions and responded with the production of roots with lower specific root length. Moreover, each litterbag contained half a gram of dried root litter and this may not have been sufficient to induce significant nutrient effects. The limited effect of decomposing litter on plant foraging response may also be due to microbial immobilisation of nutrients, which may become available to plants at the later stage of decomposition (McMahon et al. 2005; Kuzyakov and Xu 2013). The plants also had limited access to mineralised nutrients due to litter being confined to a litterbag that excluded plant roots, even though mineralised nutrients could diffuse from the litterbag into the soil solution. Future studies should consider using litter mixed directly into the soil when estimating the effects of litter decomposition on plant-soil feedback (Semchenko et al. 2017).

Neither litter chemical composition nor plant functional group explained the variation in responses to root litter of different species as we found few effects of species identity (Hypothesis 3 unsupported). The only significant effect of root litter addition that was dependent on litter species identity was detected as a change in root diameter in response to *V. cracca* litter. While root diameter in the compartment with root litter remained similar to that observed in the control treatment with an empty litterbag, the roots produced in the untreated compartment were thinner compared to the control treatment with no litter in either compartment. This suggests that the local presence of *V. cracca* litter represented unfavourable conditions for *T. repens* roots and triggered a systemic response in the form of compensatory production of thinner roots in the part of the root system not directly in contact with the litter. This finding indicates that *T. repens* can distinguish specific heterogenous environmental conditions. A similar type of response to unfavourable conditions has been demonstrated in split-root systems of *Trifolium subterraneum* where plants distinguished between nodulating and non-nodulating strains of *Rhizobium* with respect to nodule formation (Sargent et al. 1987). While several studies used seedlings with trimmed primary roots to initiate lateral growth and create split-root plants, this study employed older plants wth more developed root systems for the same purpose. The similarity in findings between our experiment and the study above suggests that both approaches initiate localised responses in plant root systems in response to a heterogeneous environment.

## Conclusions

While the legacy of plants can theoretically leave distinct effects on subsequent plant generations via litter characteristics and accumulation of specific soil biotic communities (Ke et al. 2015), there has been little focus on empirically separating the effects of plant litter and microbial soil feedback. Here we show that legacy effects mediated by soil microbiota are distinct from the effects of plant litter legacies. Moreover, heterogeneity in soil microbial legacy and litter prompted predominantly localised but also some systemic responses. These findings contribute to the growing body of studies demonstrating the ability of plants to respond locally and systemically to a diverse array of belowground cues including those related to nutrient distribution (Cahill et al. 2010), neighbours’ genetic identity (Semchenko et al. 2014) and herbivore attack (Gómez et al. 2007). Besides minor changes in root biomass, we observed changes in root morphology and nodulation. Such changes in plant traits may have consequences for nutrient cycling (Prieto et al. 2016; Roumet et al. 2016) or the strength of interactions with soil mutualists. Compensatory responses to spatial heterogeneity in soil legacies may reduce the extent of negative PSF and its potential to influence species co-existence. Therefore, these results call for the refinement of current models of PSF, which are largely based on plant responses to homogeneous soil conditions and lack a spatial dimension at the level of individual root systems.

## Electronic supplementary material

Figure S1 Split-root plants were placed in a small gap of the partitioning wall separating two compartments (each 11 × 11 × 12 cm). In the soil conditioning treatment, the treated compartment contained a mixture of 10% specifically conditioned soil in sterilised background soil, while sterilised soil in the untreated compartment was inoculated with 10% unconditioned field soil. In the litter treatment, both compartment of each pot were filled with 90% sterilised soil and 10% unconditioned field soil collected from the field. A litterbag (70 × 70 mm) containing 0.5 g of dry root litter was placed vertically into the treated compartment, 2 cm away from the outer wall. (JPEG 68 kb)
