# Supplementary figures and images for: Spatial heterogeneity in root litter and soil legacies differentially affect legume root traits
Source: Plant Soil. 2018 May 11;428(1):253–64. doi: 10.1007/s11104-018-3667-9 (PMC6435190; doi:10.1007/s11104-018-3667-9)

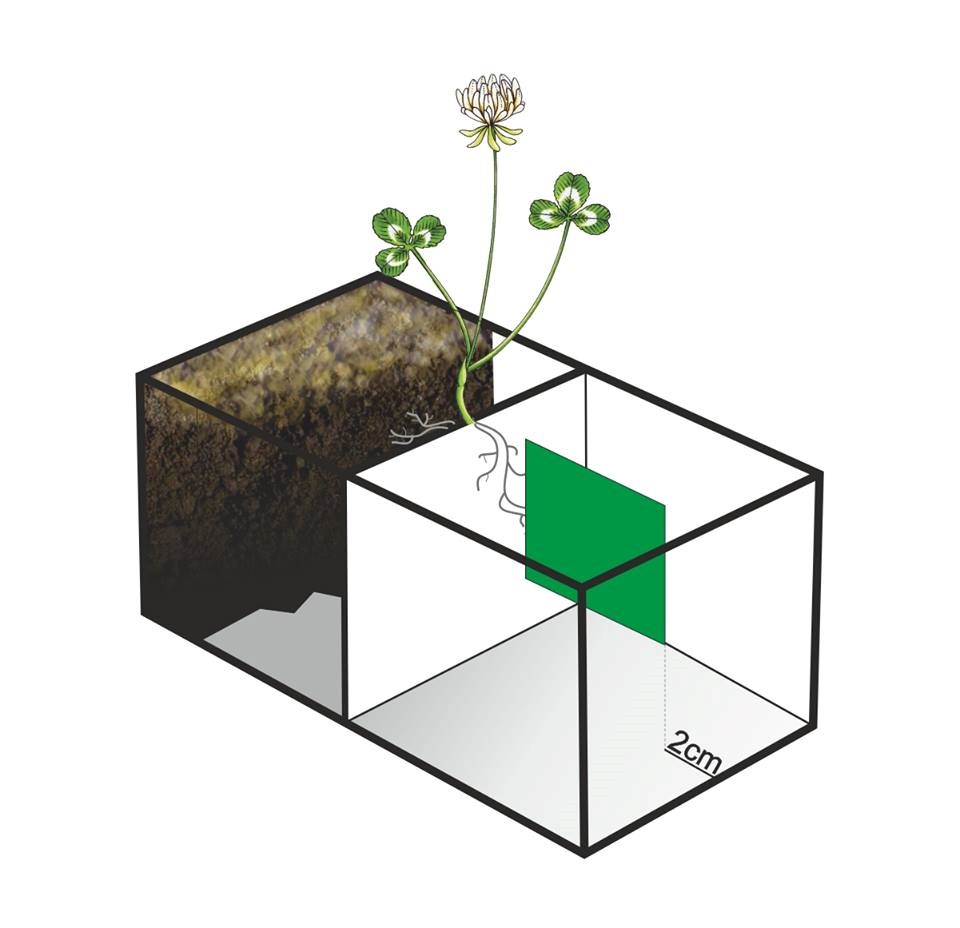

Supplement: Supplementary file 1 — Split-root plants were placed in a small gap of the partitioning wall separating two compartments (each 11 × 11 × 12 cm). In the soil conditioning treatment, the treated compartment contained a mixture of 10% specifically conditioned soil in sterilised background soil, while sterilised soil in the untreated compartment was inoculated with 10% unconditioned field soil. In the litter treatment, both compartment of each pot were filled with 90% sterilised soil and 10% unconditioned field soil collected from the field. A litterbag (70 × 70 mm) containing 0.5 g of dry root litter was placed vertically into the treated compartment, 2 cm away from the outer wall. (JPEG 68 kb) [file 11104_2018_3667_MOESM1_ESM.jpg]
